# The influence of bait on remote underwater video observations in shallow-water coastal environments associated with the North-Eastern Atlantic

**DOI:** 10.7717/peerj.9744

**Published:** 2020-08-27

**Authors:** Robyn E. Jones, Ross A. Griffin, Stephanie R. Januchowski-Hartley, Richard K.F. Unsworth

**Affiliations:** 1 College of Science, Swansea University, Swansea, UK; 2 Ocean Ecology Limited, Epney, UK

**Keywords:** Baited remote underwater video, Temperate habitats, Bait type, Bait quantity, Subtidal sediments, Fish assemblages

## Abstract

The use of baited remote underwater video (BRUV) for examining and monitoring marine biodiversity in temperate marine environments is rapidly growing, however many aspects of their effectiveness relies on assumptions based on studies from the Southern Hemisphere. The addition of bait to underwater camera systems acts as a stimulus for attracting individuals towards the camera field of view, however knowledge of the effectiveness of different bait types in northern temperate climbs is limited, particularly in dynamic coastal environments. Studies in the Southern Hemisphere indicate that oily baits are most effective whilst bait volume and weight do not impact BRUV effectiveness to any great degree. The present study assesses the influence of four bait types (mackerel, squid, crab and no bait (control)) on the relative abundance, taxonomic diversity and faunal assemblage composition at two independent locations within the North-Eastern Atlantic region; Swansea Bay, UK and Ria Formosa Lagoon, Portugal. Two different bait quantities (50 g and 350 g) were further trialled in Swansea Bay. Overall, patterns showed that baited deployments recorded statistically higher values of relative abundance and taxonomic diversity when compared to un-baited deployments in Swansea Bay but not in Ria Formosa Lagoon. No statistical evidence singled out one bait type as best performing for attracting higher abundances and taxonomic diversity in both locations. Faunal assemblage composition was however found to differ with bait type in Swansea Bay, with mackerel and squid attracting higher abundances of scavenging species compared to the crab and control treatments. With the exception of squid, bait quantity had minimal influence on bait attractiveness. It is recommended for consistency that a minimum of 50 g of cheap, oily fish such as mackerel is used as bait for BRUV deployments in shallow dynamic coastal environments in the North-Eastern Atlantic Region.

## Introduction

Baited remote underwater video (BRUV) can be used as a standardised, non-extractive technique to assess motile fauna, more specifically of fishes and fish assemblages (Cappo, Harvey & Shortis, 2006). Bait attracts individuals of different species towards the field of view of the recording camera by releasing chemical stimuli including water-soluble proteins into the surrounding water column (Wraith et al., 2013). The inclusion of bait with underwater cameras has been shown to help with overcoming the problem of low fish counts associated with fish passing un-baited systems by chance (Stobart et al., 2007) and has been utilised in both deep-sea environments (Fleury & Drazen, 2013) and shallow coastal environments (Unsworth et al., 2014).

The type and quantity of bait as well as characteristics of different species and the environment can influence or attract different motile faunal assemblages and can lead to biases in predatory or scavenging species surveyed by BRUV (Harvey et al., 2007; Yeh & Drazen, 2011; Fleury & Drazen, 2013). Studies in the southern hemisphere, over coral and rocky reef habitats, found that oily fishes such as those found from the Clupeidae and Scombridae families consistently attracted higher taxonomic diversity and abundances (Dorman, Harvey & Newman, 2012; Wraith et al., 2013; Walsh, Barrett & Hill, 2016). Equally, the plume emitted can vary depending on the physical characteristics of the bait, such as persistence, quantity, moisture content, soak time and dispersal area (Dorman, Harvey & Newman, 2012). Quantity of bait may also influence faunal abundances; more bait may attract more individuals to the camera (Hardinge et al., 2013). Attraction to a BRUV by different fauna can also be influenced by hunger levels, individual boldness, predator abundance, size of bait plume as well as hydrographic and topographic conditions (Harvey et al., 2007; Taylor, Baker & Suthers, 2013). Other considerations include the increased presence of predatory species in the vicinity of the bait potentially altering the behaviour or abundance of prey in the presence of bait (Dunlop et al., 2015; Coghlan et al., 2017).

In the North-Eastern Atlantic region, faunal assemblages associated with subtidal sediment habitats have traditionally been sampled using grabs, dredges, towed video cameras (sledge) and trawls (Kaiser et al., 2004). However, such methods can be inappropriate when in close proximity to seabed infrastructure and within or near marine protected areas, because of the methods’ destructive and mobile nature (Griffin et al., 2016; Jones et al., 2019). Challenges with sampling these dynamic environments mean that many data gaps remain for the motile fauna that inhabit these sediment habitats (Shields et al., 2011). At present, little guidance exists for BRUV deployments in the North Atlantic. With recent methodological improvements in low visibility and dynamic coastal environments (Jones et al., 2019), an opportunity exists to apply these BRUV methods to highly dynamic systems (Stobart et al., 2007; Ghazilou, Shokri & Gladstone, 2016) present in the North Atlantic region.

Here, our goal was to establish a method standardisation for BRUV deployments by determining the bait types and quantities that are best suited to shallow coastal environments associated with the North-Eastern Atlantic region. This study aims to provide an insight into bait performance and inform BRUV guidelines for future monitoring in the region. We assessed the relative abundance, taxonomic diversity and faunal assemblage composition in relation to the various bait types and quantities in two independent case study areas, Swansea Bay, United Kingdom and Ria Formosa Lagoon, Portugal. For the purpose of this study, quantitative comparisons between these two locations were not made. We hypothesized that large quantities of oily fish treatments would perform best in shallow coastal environments in the North-Eastern Atlantic region based on performance in previous bait studies. We discuss our findings from two independent case studies and provide a recommendation for future BRUV deployments in soft sediment, shallow coastal habitats in this region.

## Materials and Methods

### Site descriptions

**Figure 1:**
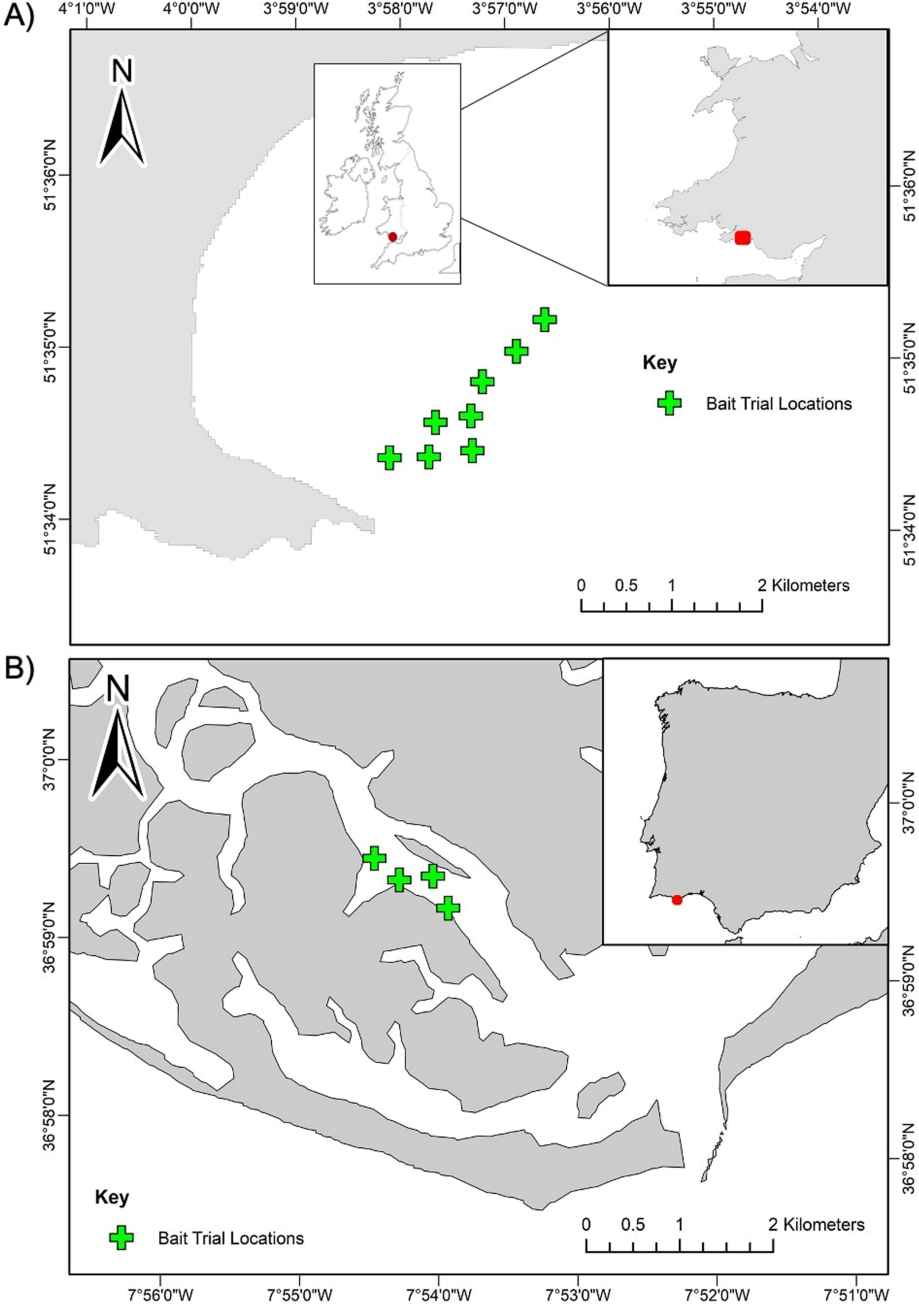
Station locations for the BRUV bait trials. (A) Swansea Bay, UK and (B) Ria Formosa lagoon, Portugal. Stations are positioned a minimum 350 m apart.

Sampling for this study was conducted at two case study locations in the North-Eastern Atlantic region: Swansea Bay, United Kingdom and Ria Formosa Lagoon, Portugal (Fig. 1). Sampling in Swansea Bay was carried out in August 2018 and in Ria Formosa Lagoon in May 2019. Swansea Bay is considered a highly dynamic environment, subject to tidal ranges of 10.5 m (Waters & Aggidis, 2016) and large tidal currents, and Ria Formosa is defined by intense morphodynamics, strong winds, and tidal rages up to 3.2 m (Ceia et al., 2010). The surveyed habitat in Swansea Bay was relatively homogeneous and consisted of subtidal sediment with fine sands and gravel patches. The surveyed habitat in Ria Formosa Lagoon was also characterised by subtidal soft sediments, but the surveyed area was more heterogeneous than Swansea Bay, with patches of seagrass beds, sandflats and saltmarshes present (Curtis & Vincent, 2005).

### Experimental design

**Figure 2:**
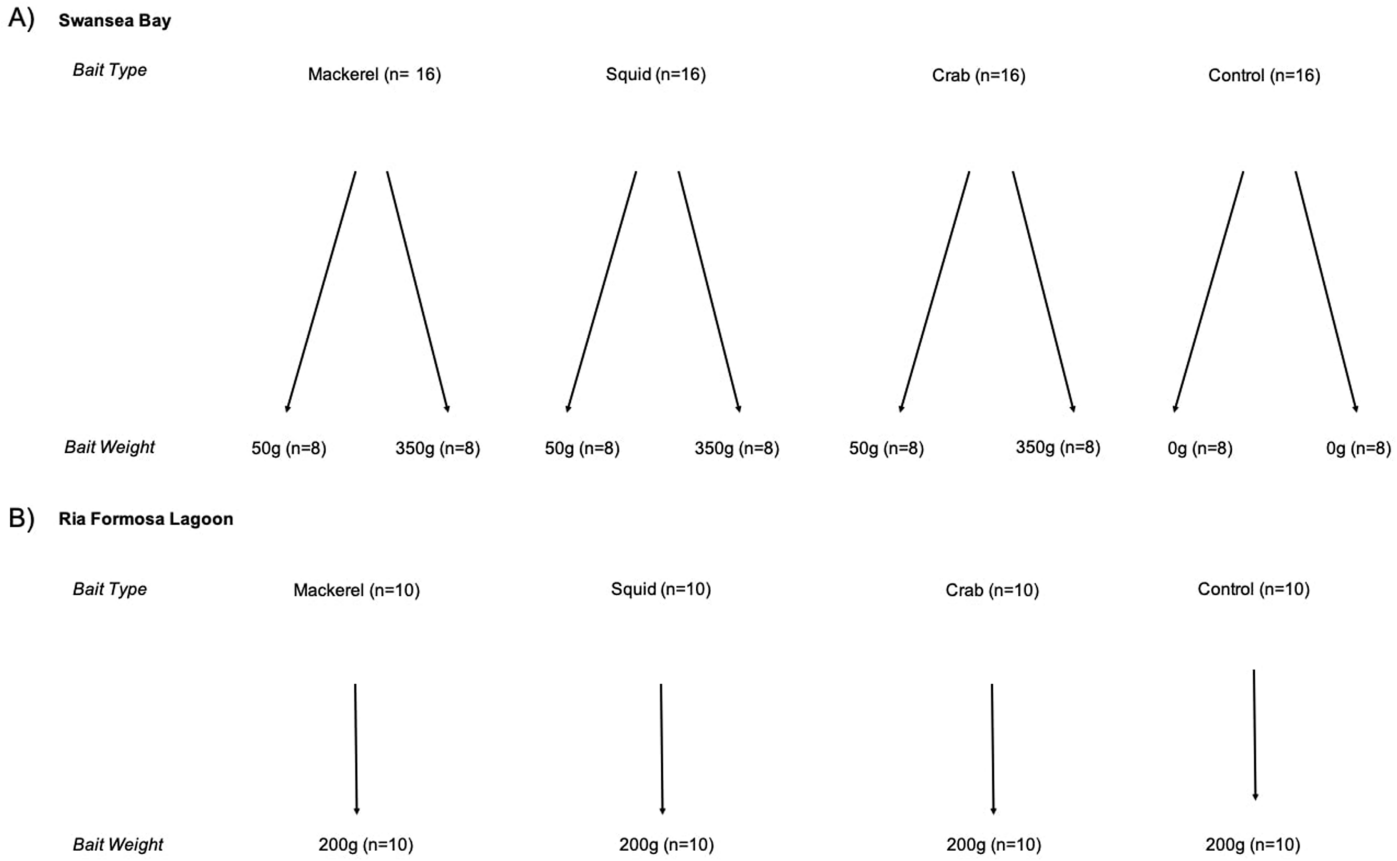
Experimental design of BRUV treatments. Replicates shown in brackets for (A) Swansea Bay and (B) Ria Formosa lagoon.

In the Swansea Bay case study, we used a two-factor design, considering both bait type (mackerel, squid, crab and no bait (control)) and weight (350 g, 50 g and no bait (control)). In the Ria Formosa Lagoon case study, we used a one-factor design with bait (mackerel, squid, crab and no bait (control)). We were unable to assess differences in bait weights in Ria Formosa Lagoon due to technical difficulties, so we used a single weight of 200 g (Fig. 2). The range of bait weights we considered in both Swansea Bay and Ria Formosa case studies are similar to those used in previous studies in the North-Eastern Atlantic Region (Unsworth et al., 2014; Peters et al., 2015; Griffin et al., 2016), but less than those most commonly reported in Australian studies (Whitmarsh, Fairweather & Huveneers, 2017). Predation induced bait depletion by high fish abundances have not previously been recorded in these study areas and is unlikely given the composition of species. Therefore, the bait weights used are reasonable given the anticipated types and abundances of species in our two case study areas.

We used eight deployment stations in Swansea Bay and four in Ria Formosa Lagoon. Each station was standardised for depth (3–10 m) and substrate type (sandy and mixed coarse sediments in Swansea Bay; soft sediments mixed with seagrass in Ria Formosa Lagoon). We deployed our stations during daylight hours (8 am–7 pm) allowing an hour between sunrise and sunset to avoid crepuscular variation in assemblages (Myers et al., 2016). Due to the modest bait weights, we determined 350 m to be sufficient distance between deployment stations to ensure independence of deployments, avoiding overlap of bait plumes and reducing the likelihood of fish moving between sites during the sampling period based on previous research (Wraith et al., 2013).

In terms of bait types, Atlantic mackerel (*Scomber scombrus*) was used at Swansea Bay and Ria Formosa Lagoon. In Swansea Bay we used Foreign Peeler Crab (*Portunus pelagicus*) and the European Common Squid (*Alloteuthis subulata*); both are commonly used by UK recreational anglers and available bait shops in the region. In Ria Formosa Lagoon, we used similar bait types, including Common Shore Crab (*Carcinus maenas*) and European Squid (*Loligo vulgaris*), which were widely available in that region. All bait types were defrosted, chopped into similar sized pieces of approximately 3 cm × 3 cm and weighed 24 h prior to sampling and placed into sealed labelled bags to retain contents.

Deployments of each treatment (a bait type and weight in Swansea Bay; a bait quantity of 200 g in Ria Formosa Lagoon) were randomly positioned across the eight stations, we ensured no replicate was deployed at the same time. Each treatment was deployed for a period of 1 h. A five mm polyvinyl chloride mesh bait bag was used to maximise dispersal, with bait replenished after every deployment.

We retrieved 51 successful deployments from Swansea Bay and 38 from Ria Formosa Lagoon. The following deployments were unsuccessful and not included in subsequent analyses: 4 × mackerel 350 g, 1 × mackerel 50 g, 2 × crab 50 g, 2 × crab 350 g, 1 × squid 50 g, 1 × squid 350 g, 1 × squid 350 g and 1 × control. Two mackerel deployments also failed in Ria Formosa Lagoon. Of the failed deployments in Swansea Bay, seven were due to low underwater visibility (meaning that the bait was not visible), two were due to a camera fault and four were due to the BRUV toppling forwards into the sediment during the deployment. The two failed deployments in Ria Formosa were due to low levels of underwater visibility.

### Sampling equipment

The mono-BRUV used during this study consisted of one Hero 4 GoPro high definition camera (GoPro, San Mateo, CA, USA) in a waterproof housing with a resolution of 1,920 × 1,080, focal length of 17.2 mm and a horizontal angle of view of 122.6° (approximately 7.3 m widest field of view). This was mounted onto an aluminium frame and weighted with 4 kg at the base for stability. A bait pole extended 65 cm in front of the camera supporting the five mm mesh bag containing the bait treatment. Each mono-BRUV system was deployed with a rope attached to a surface buoy to allow for remote deployment and recovery.

### Video analysis

All fish assemblages and motile benthic macro fauna likely to be monitored in coastal habitats using BRUV methods (Jones et al., 2019) were included in this analysis. Raw footage from each BRUV deployment was compressed to Audio Video Interleave format using Xilisoft Video/Media Converter Ultimate (www.uk.xilisoft.com) for the use of the footage in the specialist SeaGIS software Event Measure (www.seagis.com.au). We did not review any deployments where a BRUV had toppled into the sediment restricting field of view, or where the bait bag was not visible because of high levels of turbidity.

We viewed and analysed all footage for maximum number of individuals observed in a single video frame (*MaxN*) over a 1-h deployment. *MaxN* is a measure of relative abundance to avoid repeated counts of individuals (Priede, Ragley & Smith, 1994). Taxonomic diversity was calculated from the number of different species entering the camera frame during a 1-h deployment with faunal assemblage composition in each deployment recorded. Where possible, taxa were identified to species level, followed by family level if distinguishable features were not present. Organisms were identified as unknown if turbidity levels affected confidence of identification and not included further in the analysis.

### Statistical analysis

Results for the two locations were analysed as two independent case studies. We conducted all analyses in PRIMER v7 (Clarke & Gorley, 2007). Data were transformed (square root) where appropriate for count data, to reduce variance of heterogeneity.

For Swansea Bay, we assessed both total sample (combined weights under each bait type) and split sample (comparing weights within each bait type). Quantities used for the baited treatments (50 g and 350 g) differed to those used in the control treatment (0 g). A nested design was followed to allow statistical comparisons between the baited treatments and the control. For Swansea Bay, the univariate analysis consisted of a two-factor (bait and weight) permutational multivariate analysis of variance (PERMANOVA+; Anderson, 2017) using a Euclidean resemblance matrix to test for differences in relative abundance and taxonomic diversity between treatments (Table 1). In Ria Formosa lagoon, a one-factor (bait) PERMANOVA was used.

**Table 1:** Statistical analysis methods undertaken on the three variables used in the assessment of bait type and bait weight. Statistical analysis undertaken on the three variables; Relative abundance, Taxonomic Diversity and Faunal assemblage composition used in the assessment of bait type and bait weight in Swansea Bay and Ria Formosa Lagoon where appropriate.

Variable	Analysis
Swansea Bay	
Relative abundance (*MaxN*)	Univariate; Nested PERMANOVA, pairwise comparison
Taxonomic diversity	Univariate; Nested PERMANOVA, pairwise comparison
Faunal assemblage composition	Multivariate; Nested PERMANOVA, CAP, SIMPER, PERMDISP
Ria Formosa lagoon	
Relative abundance (*MaxN*)	Univariate; One-Factor PERMANOVA, pairwise comparison
Taxonomic diversity	Univariate; One Factor PERMANOVA, pairwise comparison
Faunal assemblage composition	Multivariate; Nested PERMANOVA, CAP, SIMPER, PERMDISP

For the multivariate analysis of faunal assemblage composition in Swansea Bay, a (bait and weight) PERMAONVA using a Bray–Curtis resemblance matrix was used, and a single factor PERMANOVA was used for Ria Formosa Lagoon. Principle coordinates were plotted for both locations in a constrained Canonical Analysis of Principal Coordinates (CAP) to test for differences between groups of significant factors and to visualise patterns in the data that can be hidden in unconstrained Non-metric Multidimensional Scaling plots (Anderson & Willis, 2003; Table 1). A ‘leave-one out’ cross validation analysis was undertaken to give a statistical measure of the distinctiveness of the groups (percentage allocation success) presented within the CAP plots.

All PERMANOVA tests were based on 9,999 unrestricted permutations of the raw data with significant results considered *P* < 0.05. Pairwise tests were carried out where appropriate to identify differences between treatments. Where possible, we used analysis of similarity percentages (SIMPER) to identify the main species recorded on the BRUV responsible for any differences identified between treatments. A permutational analysis of multivariate dispersions (PERMDISP) was also used to assess differences between bait types and quantities. All means are reported ±1 Standard Error (SE).

## Results

### Video analysis

From 51 BRUV deployments in Swansea Bay, we identified 130 individuals in 17 taxa and 55 individuals from sevn taxa from 38 BRUV deployments in Ria Formosa lagoon (Table 2). The greatest number of taxa were recorded using squid bait in Swansea Bay (9) and using mackerel bait in Ria Formosa Lagoon (4) (Table 2). The control (no bait) recorded the lowest number of taxa (3) in Swansea Bay, but squid had the lowest number of taxa in Ria (2) Formosa Lagoon (Table 2).

**Table 2:** Relative abundance of all taxa sampled in Swansea Bay and Ria Formosa lagoon.

Family	Species	Mean (±SE)						
		M50 g	M350 g	S50 g	S350 g	C50 g	C350 g	Control (no bait)
Swansea Bay, UK								
Arthropoda								
Paguridae	–	1.71 (±0.87)	0.50 (±0.29)	1.00 (±0.65)	1.14 (±0.51)	2.50 (±0.99)	1.33 (±0.88)	0.29 (±0.19)
Chordata								
Balistidae	*Balistes capriscus*	0.14 (±0.14)	–	–	–	–	–	–
Moronidae	*Dicentrarchus labrax*	–	–	–	0.14 (±0.14)	–	–	–
Gadidae	–	–	–	–	–	–	0.17 (±0.17)	0.21 (±0.15)
Gobiidae	–	–	–	0.14 (±0.14)	0.71 (±0.57)	0.17 (±0.17)	0.50 (±0.50)	–
Majidae	*Maja squinado*	–	–	–	0.14 (±0.14)	–	–	–
Mullidae	*Mullus surmuletus*	–	–	–	–	–	–	0.14 (±0.14)
Pleuronectidae	–	–	–	0.14 (±0.14)		0.17 (±0.17)	–	–
Rajidae	*Raja clavata*	–	–	–	0.14 (±0.14)	–	–	–
Scyliorhinidae	*Scyliorhinus canicula*	0.57 (±0.30)	–	0.14 (±0.14)	0.71 (±0.29)	0.17 (±0.17)	–	–
Sparidae	–	0.29 (±0.18)	–	–	–	–	–	–
Sparidae	*Spondyliosoma cantharus*	0.57 (±0.57)	–	–	–	0.33 (±0.33)	–	–
Triglidae	–	–	0.25 (±0.25)	0.14 (±0.14)	–	–	–	–
Triglidae	*Chelidonichthys lucerna*	0.43 (±0.20)	–	–	0.14 (±0.14)	0.17 (±0.17)	0.33 (±0.33)	–
Triakidae	*Mustelus mustelus*	0.43 (±0.20)	1.00 (±0.00)	0.29 (±0.18)	1.57 (±0.30)	0.17 (±0.17)	–	–
Mollusca								
Gastropoda	–	–	–	–	–	0.33 (±0.21)	–	–
Sepiidae	*Sepia officinalis*	–	–	–	–	0.17 (±0.17)	–	–
**Family**	**Species**	**M200 g**		**S200 g**		**C200 g**		**Control (no bait)**
Ria Formosa Lagoon, Portugal								
Chordata								
Atherinidae	*Atherina presbyter*	0.63 (±0.63)		–		0.10 (±0.10)		–
Sparidae	*Diplodus puntazzo*	–		–		–		0.10 (±0.10)
Gastropoda	–	–		–		0.10 (±0.10)		–
Mugilidae	–	2.00 (±2.00)		–		–		0.80 (±0.80)
Paguridae	–	0.75 (±0.31)		0.50 (±0.31)		0.50 (±0.17)		0.50 (±0.27)
Portunidae	–	0.13 (±0.13)		–		–		–
Rajidae	–	–		0.10 (±0.10)		–		–
Mollusca								
Gastropoda	–	–		–		0.10 (±0.10)		–

**Note:**

The mean (± 1SE) relative abundance (*MaxN*) of all taxa sampled during BRUV deployments in Swansea Bay (above) and Ria Formosa lagoon (below) using different bait treatments (M = Mackerel; S = Squid; C = Crab).

### Swansea Bay

#### Relative abundance

The PERMANOVA test for relative abundance (*MaxN*) showed statistical differences between the four bait type treatments in Swansea Bay (*F*_3,44_ = 4.5051, *P* = 0.01; Table 3), but not for the bait quantities within the different bait type treatments (*F*_3,44_ = 2.948, *P* = 0.05; Table 3). A pair-wise test identified that the control treatment (no bait) recorded a significantly lower relative abundance compared to all three baited treatments. No differences in relative abundance between the three bait types were observed.

**Table 3:** PERMANOVA statistical analysis for *MaxN*, taxonomic diversity and faunal assemblage composition.

Source	df	MS	*Pseudo-F*	*P* (perm)	Unique perms
*MaxN*					
Swansea bait type	3	3.5293	4.5051	**0.01**	9,950
Swansea bait quantity (bait type)	3	2.3094	2.948	0.05	9,954
Residual	44	0.7833			
Total	50				
Ria Formosa lagoon bait type	3	1.2029	1.3503	0.2778	9,269
Residual	34	0.89082			
Total	37				
*Taxonomic diversity*					
Swansea bait type	3	2.7439	7.8532	**<0.001**	9,949
Swansea bait quantity (bait type)	3	1.5970	4.5706	**0.01**	9,948
Residual	44	0.3494			
Total	50				
Ria Formosa lagoon bait type	3	0.25788	0.75963	0.5297	971
Residual	34	0.33948			
Total	37				
*Faunal assemblage composition*					
Swansea bait type	3	4,651.7	4.758	**<0.001**	9,929
Swansea bait quantity (bait type)	3	3,038.4	3.039	**0.001**	9,928
Residual	44	978.31			
Total	50				
Ria Formosa lagoon bait type	3	563.82	0.78809	0.5969	9,933
Residual	34	715.42			
Total	37				

**Note:**

PERMANOVA of *MaxN* (relative abaundance), Taxonomic diversity and faunal assemblage composition in Swansea Bay, South Wales and Ria Formosa lagoon, Portugal. Bold values *P* < 0.05.

**Figure 3:**
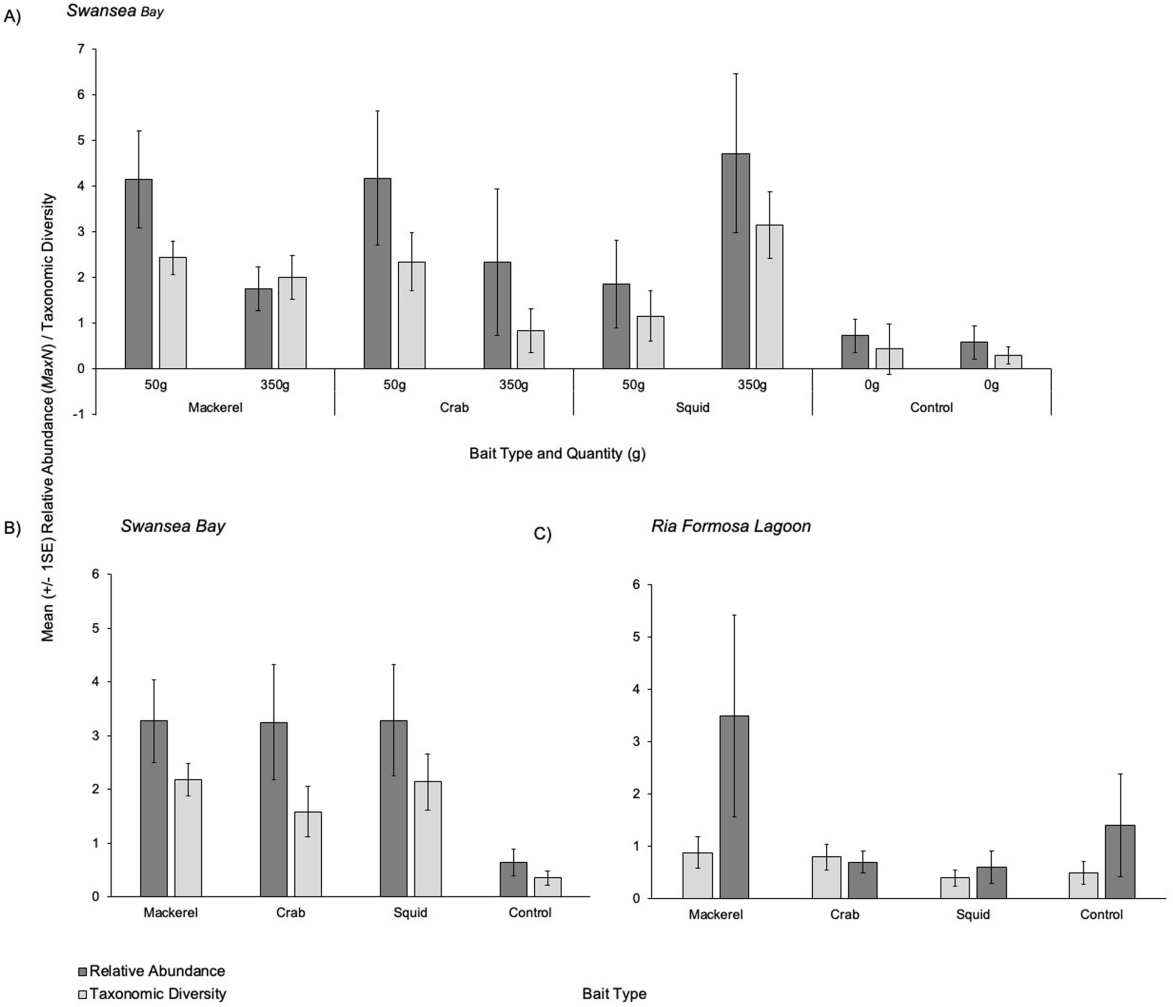
Mean (±1 SE) relative abundance/taxonomic diversity. (A) For the different bait and weight treatments in Swansea Bay (B) for the combined bait type treatments in Swansea Bay (C) for the bait type treatments in Ria Formosa lagoon.

Overall patterns showed that all three baited treatments presented similar mean relative abundances (*MaxN*) captured on camera with 3.29 (±1.03), 3.27 (±0.76) and 3.25 (±1.07) individuals for squid, mackerel and crab respectively (Figs. 3A and 3B). The control (no bait) treatment presented a mean of 0.64 (±0.25) individuals. When splitting the bait treatments by weight, 350 g of squid had the highest mean relative abundance (4.71 ±1.74) (Fig. 3A). Smaller quantities (50 g) of crab and mackerel recorded similar abundances of 4.17 ±1.47 and 4.14 ±1.06 respectively.

#### Taxonomic diversity

The PERMANOVA test for differences in taxonomic diversity in Swansea Bay showed statistical differences between the four bait type treatments (*F*_3,44_ = 7.8532, *P* =< 0.001; Table 3) and the bait quantities within the different bait type treatments (F_3,__44_ = 4.5706, *P* = 0.01; Table 3). A pair-wise test identified that the control treatment (no bait) recorded a significantly lower relative abundance compared to all three baited treatments. No significant differences between the three bait types for taxonomic diversity were observed. A second pairwise test for bait quantities identified that only 50 g of squid recorded significantly less taxonomic diversity compared to other baited deployments using 350 g (Post hoc: *t* = 2.59, *P* = 0.03).

Overall patterns showed that mackerel and squid presented a similar mean taxonomic diversity captured on camera with 2.18 (±0.30) and 2.14 (±0.52) individuals respectively (Figs. 3A and 3B). When splitting each bait type treatment by weight in Swansea Bay, 350 g of squid recorded the highest mean taxonomic diversity (3.14 ±0.74) followed by 50 g mackerel treatment with a mean of (2.43 ± 0.37); Fig. 3A.

#### Faunal assemblage composition

The PERMANOVA test of faunal assemblage composition in Swansea Bay showed a significant treatment effect for bait type (*F*_3,44_ = 4.758, *P* =< 0.001; Table 3). A pair-wise test identified that significant differences were present between the control treatment and all three baited treatments. Statistical differences were also identified between crab and the squid and mackerel treatments (Post hoc; *t* = 1.74, *P* = 0.03 and *t* = 1.67, *P* = 0.04 respectively). No statistical differences were present between mackerel and squid. For bait quantities, statistical differences in faunal composition were present (*F*_3,44_ = 3.039, *P* = 0.001; Table 3) between the 50 and 350 g treatments within the squid bait treatment (Post hoc; *t* = 2.04, *P* = 0.02) only.

**Figure 4:**
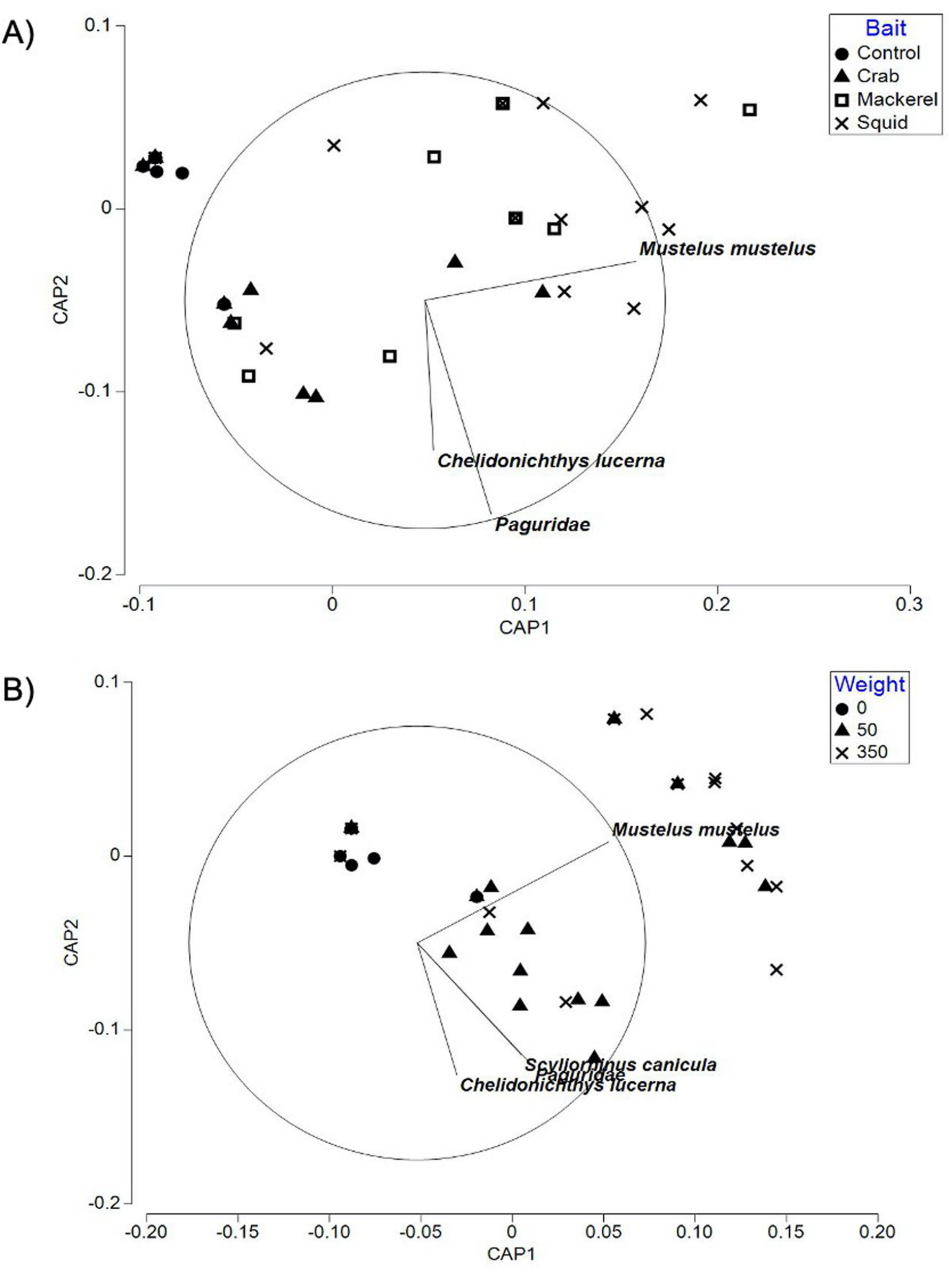
CAP ordination for fish assemblages sampled by BRUV deployments. (A) Swansea Bay for the four bait type treatments (B) Swansea Bay for the three bait quantity treatments. Vector lines refer to strongly correlated faunal assemblages (>0.6) with the direction and length of line indicating the direction and strength of correlation in relation to the first and second CAP axes.

The CAP plot for bait type in Swansea Bay (Fig. 4A) showed patterns in bait type identified in the PERMANOVA. Control deployments were separated out from the majority of the squid and mackerel deployments along CAP axis 1 in the negative values with crab deployments occasionally overlapping. Baited deployments ranged into both the positive and negative values of CAP axis 1. With the exception of the control treatment (85.71%), the baited groups had a relatively low ‘leave one out’ allocation success. Out of the 51 samples, only 21 of the deployments were correctly classified. There was a mis-classification error 58.82%, indicating that overall similar faunal assemblages were sampled using baited deployments when compared to unbaited deployments.

Similarly, the CAP plot for bait quantities (Fig. 4B) also showed the control treatment to be separated out from the majority of the baited treatments along CAP axis 1. All taxa with a correlation greater than 0.6 to either CAP axes were correlated towards the baited treatments. The ‘leave one out’ allocation showed a good allocation success for the control treatment (85.71%) but a low allocation success for the 50 g (45.00%) and 350 g (58.82%) treatments. Out of the 51 samples, 31 of the deployments were correctly classified with a mis-classification error of 39.22%.

A test of PERMDISP between treatments in Swansea Bay identified a statistically significant variation between bait type treatments (bait type; 3, 47 *F* = 5.207, *P* = 0.01) and bait quantities (bait weight; 2, 48 *F* = 12.531, *P* = <0.001), suggesting a significant spread of these results around the spatial mean. Deployments of the control (34.42 ± 2.96) and 50 g of bait treatments were identified to be the most variable (38.40 ± 1.63). This variation may be attributed to the sparse nature of faunal assemblages found on sediments in these environments.

A SIMPER analysis (Table 4) identified abundances of Paguridae, Gadidae, *M. mustelus* and *S. canicula* as the main organisms responsible for differences between the three bait types and the control treatment.

**Table 4:** SIMPER statistical analysis in Swansea Bay. SIMPER analysis in groups outlined by PERMANOVA showing the organisms which most contributed (>70% cumulative contribution) to the observed differences among bait type treatments in Swansea Bay.

Species	Av. abun.	Av. abun.	Av. diss.	Diss./SD	Contrib. %	Cum %
Av. diss.: 92.37	Control	Crab				
Paguridae	0.20	1.01	42.21	1.24	45.73	45.73
Gadidae	0.17	0.08	17.06	0.50	18.49	64.22
*M. surmuletus*	0.10	0.00	5.21	0.27	5.64	69.87
Gobiidae	0.00	0.23	4.77	0.48	5.16	75.03
Av. diss.: 93.72	Control	Mackerel				
*M. mustelus*	0.00	0.64	33.91	0.96	35.42	35.42
Paguridae	0.20	0.77	25.50	1.06	26.63	62.06
*C. lucerna*	0.00	0.27	8.22	0.56	8.58	70.64
Av. diss. :95.36	Control	Squid				
*M. mustelus*	0.00	0.75	31.21	1.04	32.46	32.46
Paguridae	0.20	0.70	24.36	0.97	25.33	57.79
*S. canicula*	0.00	0.39	14.55	0.60	15.13	72.91
Av. diss.: 85.44	Crab	Squid				
Paguridae	1.01	0.70	25.47	1.06	29.89	29.82
*M. mustelus*	0.08	0.75	21.05	0.86	24.66	54.48
*S. canicula*	0.08	0.39	10.66	0.57	12.48	66.97
Gobiidae	0.23	0.29	6.42	0.68	7.52	74.48
Av. diss.: 84.03	Crab	Mackerel				
Paguridae	1.01	0.77	23.30	1.13	27.94	27.94
*M. mustelus*	0.08	0.64	23.05	0.79	27.64	55.58
*C. lucerna*	0.20	0.27	7.740	0.64	9.28	64.86
*S. canicula*	0.08	0.31	6.390	0.62	7.67	72.52

### Ria Formosa lagoon

#### Relative abundance

The PERMANOVA test for relative abundance in the Ria Formosa Lagoon showed no statistical differences between the four bait treatments (Fig. 3C) (*F*_3,37_ = 1.3503, *P* = 0.2778; Table 3). Overall patterns show that mackerel recorded the highest relative abundance (*MaxN*) with a mean of 3.50 (±1.93) individuals. Squid had the lowest mean relative abundance (0.60 ±0.31).

#### Taxonomic diversity

The PERMANOVA test for differences in taxonomic diversity showed no statistical differences between the four bait types (*F*_3,37_ = 0.75963, *P* = 0.5297; Table 3). Similar to mean relative abundance, mackerel recorded the highest taxonomic diversity in Ria Formosa Lagoon with 0.88 (±0.30) taxa. The squid treatment recorded the lowest taxonomic diversity with a mean of 0.40 (±0.16) taxa.

#### Faunal assemblage composition

**Figure 5:**
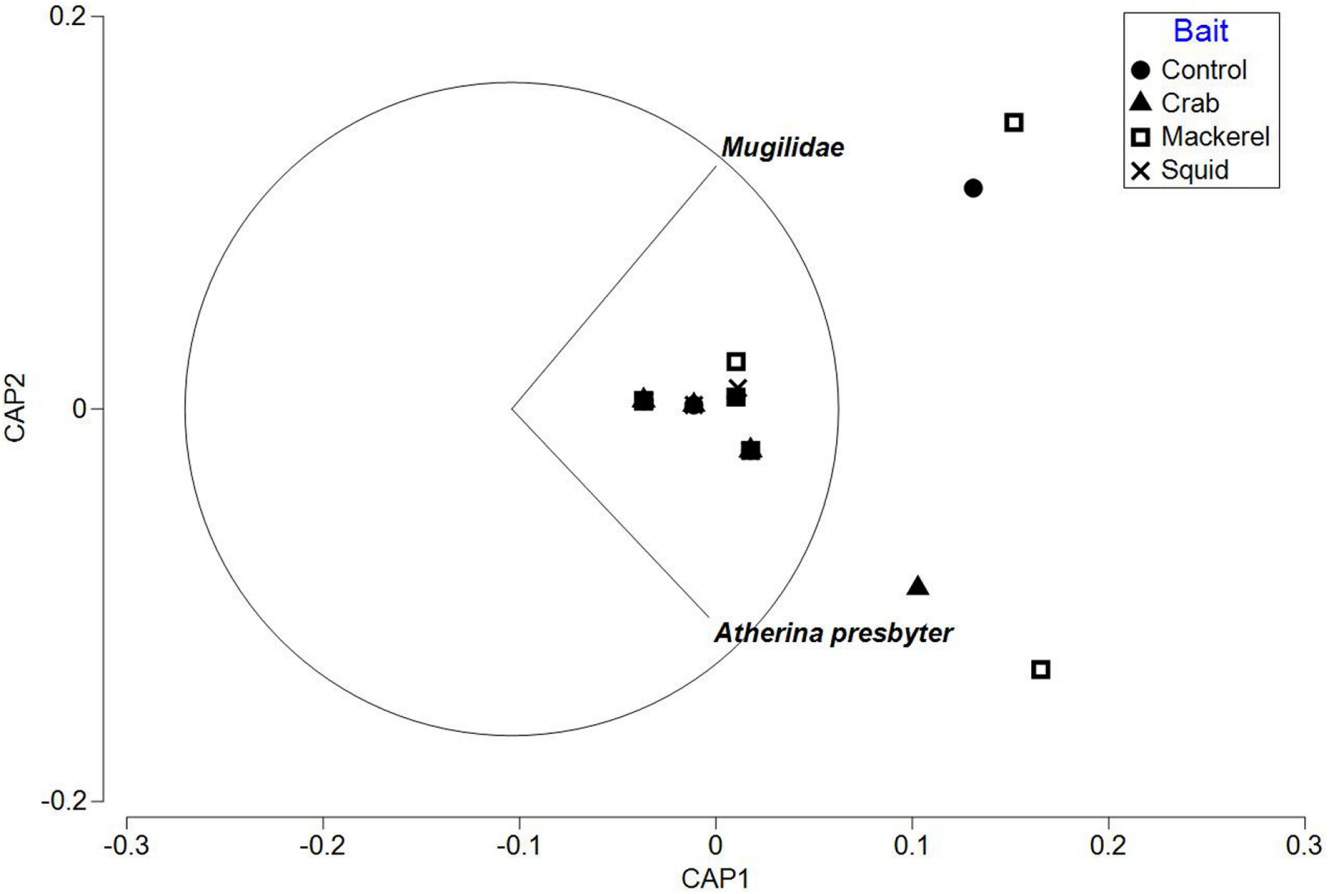
CAP ordination for fish assemblages sampled by BRUV deployments in Ria Formosa lagoon for the four bait types. Vector lines refer to strongly correlated faunal assemblages (>0.6) with the direction and length of line indicating the direction and strength of correlation in relation to the first and second CAP axes.

The PERMANOVA test on relative abundance (*MaxN*) showed no significant treatment effect of bait type on faunal composition (*F*_3, 37_ = 0.78809, *P* = 0.5969; Table 3). The CAP plot for bait type (Fig. 5) confirms the PERMANOVA results with no clear distinction between treatments along CAP axes.

The ‘leave one out’ allocation presented a low allocation success (0.00%) for all bait treatments with the exception of the squid treatment (60.00%). Out of the 38 samples, only 10 of the deployments were correctly classified with a misclassification error of 73.68%. The PERMDISP test between bait types showed no statistically significant different variation between treatments (bait type: 3, 34 *F* = 2.018, *P* = 0.301).

## Discussion

This study found that baited deployments, on average, attracted higher relative abundance and taxonomic diversity of marine fauna compared to unbaited deployments, but that these were not found to be statistically different between bait types. In Swansea Bay, statistically higher relative abundances and taxonomic diversity were recorded for baited deployments compared to unbaited deployments. Faunal assemblage analysis also in Swansea identified differences in composition between baited and unbaited treatments as well as between the crab treatment and the mackerel and squid treatments. We discuss these findings and wider implications for future studies using BRUV deployments to monitor underwater species diversity in the North-Eastern Atlantic Region.

Our findings correspond to previous studies comparing baited and un-baited camera deployments (Dorman, Harvey & Newman, 2012; Bernard & Götz, 2012; Wraith et al., 2013; Hannah & Blume, 2014) where the presence of bait has been found to both increase similarity between replicates and detect changes in fauna between habitat types (Whitmarsh, Fairweather & Huveneers, 2017). Scavenging and opportunistic species (Lyle, 1983; Saïdi et al., 2009; Stagioni, Montanini & Vallisneri, 2012) including Paguridae, *M. mustelus, S. canicula* and *C. lucerna* were heavily correlated with baited camera deployments. Increased numbers of these individuals also influenced statistical differences between baited and unbaited treatments. Studies applying BRUV methods to deep-sea environments have found similar shifts in community composition when using bait in camera deployments with the abundance of scavenging species increasing in the presence of bait (Yeh & Drazen, 2011). Differences in faunal composition were also noted between crab and mackerel and squid, however, with further analysis only showing Paguridae as having higher abundances when using crab, mackerel and squid were considered better for faunal coverage.

At both study locations, low numbers of relative abundance and taxonomic diversity were observed for the majority of species, and this could have impacted our results. BRUV performance may have been influenced by variables such as the distribution of species over large spatial areas at both locations. Subtidal soft sediment habitats such as those surveyed in Swansea Bay and Ria Formosa Lagoon provide far less structural heterogeneity when compared to reef type habitats and can remain homogenous over large areas (Syms & Jones, 2004). The distribution of organisms on subtidal soft sediment habitats often depends on factors such as food availability, disturbance and seabed complexity which, in large habitats, may influence a large spatial distribution of individuals (McCormick, 1995; Parsons et al., 2014). Furthermore, underwater visibility was relatively low at both locations which may have limited the relative abundance and diversity of species recorded during deployments. Seven and two of the failed deployments in Swansea Bay and Ria Formosa Lagoon respectively were due to very high levels of turbidity obscuring the bait. The large tidal ranges observed in Swansea Bay alongside its shallow nature equate to a large amount of sediments suspended into the water column (Collins & Banner, 1980). In Ria Formosa Lagoon high turbidity levels are attributed to agricultural run-off and sewage (Newton & Mudge, 2005).

Relative abundance and taxonomic diversity were lower than expected in the Ria Formosa Lagoon in particular, based on previous monitoring studies using seine netting techniques at this location (Ribeiro et al., 2008). The variability of anthropogenic activity in this instance could have additionally influenced the performance of our BRUV deployments in this study location. There was a notable difference in motorized boat traffic between the two survey locations, with Ria Formosa Lagoon harbouring higher numbers of small vessels over a small spatial area (Correia et al., 2015). Previous studies showed that anthropogenic noise associated with recreational motorized boat activity has the potential to impact fish movements and behaviour (Slabbekoorn et al., 2010; Nichols, Anderson & Širović, 2015; Roberts, Pérez-Domínguez & Elliott, 2016). Further research into the impacts of anthropogenic noise on BRUV performance would provide an interesting insight into this.

Contrary to our hypothesis, more bait (e.g. 350 g) did not perform better than less bait, in terms of mean relative abundance observed for baited treatments in Swansea Bay. However, 350 g of the squid treatment presented a significantly higher taxonomic diversity compared to its 50 g counterpart during deployments suggesting that a higher quantity of squid is required to gain higher values of diversity compared to other baits. Findings in other coastal studies have found that BRUV deployments with higher bait quantities do not necessarily improve bait performance in deployments (Hardinge et al., 2013). Our finding contradicts previous findings for bait quantities used in traps (Miller, 1983; Cyr & Sainte-Marie, 1995) and plume models in deep sea environments (Sainte-Marie & Hargrave, 1987), where higher bait quantities produced higher relative abundance of scavenging amphipods. This suggests that bait quantity is likely to have a different effect depending on faunal assemblage sampled that is swarms of amphipods compared to larger scavengers such as fish or crabs. Taylor, Baker & Suthers (2013) found that, in dynamic coastal environments, bait plume penetration and dispersal could be primarily driven by tidal currents influencing the probability of assemblages locating relevant attractants (Hill & Wassenberg, 1999; Heagney et al., 2007; Stiansen et al., 2010). Although environmental parameters were not measured during this study, our findings support those of previous studies in dynamic coastal environments, which suggest that external environmental factors are more likely to influence bait performance compared to bait quantity.

Various quantities of bait have previously been used in BRUV studies globally ranging from 50 g to >2 kg (Whitmarsh, Fairweather & Huveneers, 2017). Compared to Australian BRUV studies where records show that up to 1 kg of bait can be consumed or removed during an hour deployment (Dorman, Harvey & Newman, 2012), minimal bait depletion was observed in Swansea Bay for both bait weights tested. This may have also influenced the small differences in relative abundance and taxonomic diversity observed further suggesting that assemblages are equally attracted to the bait throughout the deployment regardless of the bait quantity used (Harvey et al., 2007). In areas where scavenging rates are higher, we expect bait quantity to be more important. Similarly, the majority of 200 g bait also remained in the bait bag after all 1-h BRUV deployments in Ria Formosa lagoon for all three bait types.

### Recommendations

Coastal habitats assessed during this research comprised primarily of subtidal soft sediments in dynamic environments less than 10 m depth. Although, no one individual bait type provided a statistically higher relative abundance or taxonomic diversity, smaller quantities of mackerel and crab (e.g. 50 g), were found to produce similar values of relative abundance and taxonomic diversity as larger quantities (e.g. 350 g) of squid in Swansea Bay. It is recommended for consistency and standardisation that when implementing BRUV methods in these environments, bait use should include locally sourced oily fish such as mackerel. Compared to other bait types such as crustaceans and cephalopods, oily fish is considered a much cheaper alternative and is readily available in both local angling shops and supermarkets. Following methods used in previous studies, best practice for bait is to defrost for at least 24 h prior to deployments in order to generate a greater aroma and bait plume once in the water (Dorman, Harvey & Newman, 2012). To maximise effectiveness, bait should be replenished after each deployment as an increased soak time has been found to reduce bait quality over time (Løkkeborg & Johannessen, 1992). We suggest that for deployments in this region, minimum quantities of 50 g are sufficient for attracting organisms to the camera field of view.

## Conclusions

Statistically higher relative abundance and taxonomic diversity were recorded for baited camera deployments compared to unbaited deployments in Swansea Bay. The influence of bait quantity was only present for the squid bait type. Statistical differences were also found for faunal assemblage composition between bait types in this study area. Mackerel and squid recorded similar abundance values for scavenging species such as *M. mustelus* and *S. canicula* that were statistically greater than those returned by crab or control treatments where records of these species were minimal. We found no statistical evidence for a single bait type influencing relative abundance, taxonomic diversity or faunal composition in the Ria Formosa lagoon study area potentially due to the lower numbers of abundance and diversity recorded in this location.

## Additional Information and Declarations

### Data Availability

The following information was supplied regarding data availability: Data for this study is available at the Pangea Earth and Environmental Science Database: Citation: Jones, Robyn E. (2019): Bait Trials North Atlantic Region. PANGAEA. DOI 10.1594/PANGAEA.908178.
